# Humoral and cellular immune response induced by antigenic protein of *Sarcoptes scabiei* var. *caprae*

**DOI:** 10.14202/vetworld.2018.819-823

**Published:** 2018-06-19

**Authors:** Nunuk Dyah Retno Lastuti, Wiwik Misaco Yuniarti, Poedji Hastutiek, Lucia Tri Suwanti, Dony Chrismanto

**Affiliations:** 1 Department of Parasitology, Faculty of Veterinary Medicine, Universitas Airlangga, Surabaya, Indonesia; 2 Department of Veterinary Clinic, Faculty of Veterinary Medicine, Universitas Airlangga, Surabaya, Indonesia; 3 Study Programme of Animal Health, Faculty of Vocation, Universitas Airlangga, Surabaya, Indonesia

**Keywords:** antigenic protein, CD4, CD8, immunoglobulin G, *Sarcoptes scabiei* var*. caprae*, toll-like receptor-9

## Abstract

**Aim:**

Scabies is one of the most important diseases in goats and caused by a complex hypersensitivity process that involves both humoral and cell-mediated immune responses. This phenomenon shows that the variety of *Sarcoptes scabiei* has different characteristics of specific antigenic protein or different immune-dominant. This research aims to detect the humoral and cellular immune response of rabbits which were immunized with the protein of *S. scabiei* var. *caprae*.

**Materials and Methods:**

This research was done as follows, identification and collection of *Sarcoptes scabie*i var *capra*e from goat infected with scabies, separation of protein antigen from *S. scabie*i mites with ultrasonic sonicator, measurement of protein content with spectrophotometry, rabbit injection with 500 μg dose of antigen protein which was repeated 5 times (5x booster) every 2 weeks. Measurement of IgG titer using indirect ELISA, whereas to detect the expression of cellular immune response (TLR-9, CD4, and CD8) using Direct Immunofluorescence assay.

**Results:**

Based on the statistical analysis, it showed that there was a significant enhancement (p<0.05) for optical density value or antibody titer and cellular immune response was shown by TLR-9, CD4, and CD8 expression in rabbit T lymphocytes which appear yellow to green fluorescent color using fluorescence microscope. The amount of fluorescence T lymphocytes showed a significant difference (p<0.05) between the control and various boosters.

**Conclusion:**

Antigenic protein of *S. scabiei* var. *caprae* contains ligands, which are involved in the pathogen-associated molecular pattern that has an ability to induce humoral and cellular immune response in rabbit. Specifically, that TLR-9 is not only involved in innate immunity but also in adaptive immunity and can be used as alternative adjuvant development research.

## Introduction

Sarcoptic mange is one of the most economically important diseases in goats in Indonesia, but the control of this ectoparasite is limited [1]. An increasing number of treatment failures is being reported because of the drug resistance. Vaccination is an attractive ecological alternative to the use of acaricides for the parasite control. However, effective antiparasite vaccines against sarcoptic mange have not yet been developed [2].

At present, it is considered as an emerging/re-emerging parasitic disease that threatens human and animal healthy globally [3]. Since the prevalence of the disease in human and animals is very high, the economic losses caused by the disease are enormous. The development of a specific vaccine would be a sustainable option for the control of this disease [4].

Vaccine development requires preliminary research through immunogenic protein exploration of *Sarcoptes scabiei* which is isolated from goats. Previous studies have shown that *S. scabiei* var. *caprae* contains antigenic proteins with molecule weight 205.8 kDa, 57.3 kDa, 43.0 kDa; these three proteins have antigenic characteristics which are probably involved in scabies pathogenesis in goats [5].

Immunogenic protein can work maximally as a vaccine when it can stimulate T cells activation together with co-receptor CD4 and CD8, which plays a role in the immune system activation. CD4+ T cells will help B cells to produce antibodies and also it helps phagocytosis to destroy ingested microbes, while CD8+T cells will kill intracellular microbes [6-9]. It has been known that toll-like receptor (TLR) in lymphocytes act as an essential signal to regulate lymphocytes activation and proliferation, produce antibodies, and regulate antigen presentation. TLR stimulation through microbial product initiates signaling pathways which activate not only the innate immunity but also the adaptive immunity [6,10-12]. To investigate that TLR plays a role in adaptive immunity, it requires research toward *S. scabiei* protein immunized in rabbits to detect cellular immune response and TLR-9 as markers. TLR-9 has been developed by some researchers as vaccine adjuvant containing synthetic oligodeoxynucleotide to fight infectious diseases, allergies, and cancers [13-16].

The aim of the study was to detect humoral and cellular immune response of rabbits which were immunized with *S. scabiei* var*. caprae* protein. Increasing optical density (OD) value or antibody titer (immunoglobulin G [IgG]) as a parameter of humoral immune response and TLR-9, CD4, and CD8 as a parameter of cellular immune response that could be used preliminary study for the development of subunit vaccine as an alternative for scabies prevention on goats in Indonesia.

## Materials and Methods

### Ethical approval

The entire research was conducted appropriately following the ethics in using experimental animals and has been approved by the Ethics Commission of the Faculty of Veterinary Medicine, Universitas Airlangga, No: 630-KE.

### Isolation of *S. scabiei* mites and rabbits immunization

*S. scabiei* mites (adults, nymphs, and larvae) were isolated from domestic goats that showed clinical signs of scabies such as thickening of the skin, crust formation, and alopecia on the area around eyes, ears, mouth, and legs [5,6,17]. After the identification based on key identification from Soulsby [17], mites isolation was required for rabbits immunization by these following steps: Mites (around one thousand mites) were put into a Petri dish and mixed with phosphate-buffered saline (PBS) solution and strained to get the result free of skin crust. The result was washed with PBS solution and centrifuged at 3000 rpm for 10 min. The washing process was performed at least 3 times, to get mites free of dirty materials that were carried from the scraping process.

The deposited mites would be formed as pellets and kept in a freezer at −80°C, to be processed into homogenized protein [5]. The pellets (mites) with homogenizing medium: 100 mM Tris-HCL buffer pH 7.4, 100 mM NaCl, 5 mM EDTA, 5 mM MgCl_2_ [18] were sonicated at 30 K Hz and this step was repeated for 16 times. Every sonication step lasts for 4 min with a break time of 2 min [5]. The sonication result solution was centrifuged at 16,000 rpm for 5 min; then, the protein concentration of the resulting supernatant was measured during visible spectrophotometer with 595 nm wavelength.

This research used six rabbits, 4 months old and prepared for the immunization trial at the laboratory of experimental animals, Faculty of Veterinary Medicine, Universitas Airlangga. Each of experimental rabbits was injected with 500 µg *S. scabiei* protein (0.3 ml) and added Freund adjuvant complete (Sigma, USA) with ratio 1:1. Every 2 weeks, 5 times the injection was performed with the same protein as a booster. Before the first injection, about 10 ml of rabbit blood was taken for TLR-9, CD4, and CD8 examination as preliminary data (control) and whole blood examination was conducted at the end of first booster until the fifth booster.

### Examination of humoral immune response (IgG) with Indirect ELISA

To assess the humoral immune response (IgG), blood samples were collected from the marginal ear vein before immunization (control), on the 14^th^ day (1^st^ booster), the 28^th^ day (2^nd^ booster), the 42^th^ day (3^rd^ booster), the 56^th^ day(4^th^ booster), and the 70^th^ day (5^th^ booster). Serum samples were obtained from blood and stored at −20°C until use.

High-binding microtiter plates (BIO-RAD, USA) were coated overnight at 4°C with 100 µl of antigen solution of *S. scabiei* (10 µl/ml antigen in PBS) in each, then washed with 200 µl PBS-T20 for 3 times. After that, blocking with 4% skim milk (Maxcream, Indonesia) in PBS-Tween as much as 200 µl/well and incubated at 37°C for an hour, then washed with PBS-T20 for 3 times. Rabbits’ serum from immunization result was added and tested at 1/200 dilution as much as 100 µl for each well. This stage was performed in twice.

For antibody measurement standard, antibody dilution was done that the negative control antibody titer had been known at dilution of 1/200 up to 1/204.800 and added 100 µl for each well and then incubated at 37°C for an hour and washed. The next step was the addition of conjugate IgG anti-rabbit (Promega Corporation, USA) with 1/4000 dilution as much as 100 μl per well and incubated at 37 ºC for one h. Then, the plate was washed and added substrate pNPP into substrate buffer (diethanolamine 1 mg/ml) as much as 100 µl for each well. Incubation was done in a dark room at room temperature for 15-45 min. The reaction was stopped by adding 3N NaOH solution for 50 µl in each well, and the plate was read using ELISA reader (BIO-RAD, USA) with wavelength of 405 nm.

The ELISA reading was done as these following steps: OD value standard (positive or negative) was sorted by the maximum to the minimum OD value. As the negative control limit, it was taken OD value at dilution 1/200. The highest dilution of positive control antibody that is still showing positive value based on cutoff value (2 times the average negative control) is an antibody titer. Then, it was made a statistic range from antibody standard, for example, the maximum OD range >3.171 has antibody titer 204.800, between 2.999 and 3.171 has antibody titer 102.400, and so on up to the zero titer. For measuring sample OD value, put sample OD value on the titer range below. Antibody titer showed a negative result if the ELISA reading result between control rabbits and immunized rabbits obtained a similar OD value. Antibody titer showed a positive result if OD value from immunized rabbits was higher or at least 2 times larger than OD value from control rabbits [19].

### Immunofluorescence technique for TLR-9, CD4, and CD8 examination

For the examination of TLR-9, CD4, and CD8, the isolation of PBMC in T cells was performed based on Boyum method (1968) with some modifications [19], as these following procedures: 5 ml whole blood and washed with 10% PBS, centrifuged at 1600 rpm, and temperature 10°C for 10 min, next the undercoat to be put on Ficoll isopaque. The mixture containing blood and Ficoll isopaque was centrifuged at 1600 rpm and temperature 10°C for 10 min. The resultant buffy coat was separated and washed with Tween PBS. TLR-9 examination was performed as these following stages, buffy coat, and 300 µl MEM (Sigma, USA) incubated at 37°C for an hour, the solution was fixed by absolute methanol, and blocked by PBS and 1% fetal calf serum for 15 min, next the solution was washed using Tween PBS and there was added the first antibody of monoclonal antibody TLR9-fluorescein isothiocyanate (FITC) labeled (IMGENEX Corp). The solution added 1% fetal calf serum and FITC conjugate. The result was examined by fluorescence microscope using 400x magnification, to find out whether any yellow-to-green fluorescence color from T cells expressing TLR-9. If there is fluorescence light from T cells showing an activated immune response, then the calculation is conducted toward the number of fluorescent T cells on 20 µl solution.

Examination of CD4 and CD8 was performed as these following: buffy coat was centrifuged at 1700 rpm for 5 min, the supernatant was removed, 30 µl 70% methanol was added to the pellet and make it into suspension. Next, the suspension was incubated for 5 min at room temperature and centrifuged at 10.000 rpm for 5 min. The supernatant was removed, washed with Tween PBS once, and centrifuged at 10.000 rpm for 5 min, then added 200 µl PBS and make it into suspension. 10 µl of solution was taken, and put into a tube, added 10 µl Monoclonal Mouse Anti-Human CD4, and Clone MT310 (DakoCytomation, Denmark), Monoclonal Mouse Anti-Human CD8, Clone DK25 (DakoCytomation, Denmark). Make the solution into suspension and incubated in dark room at 4°C for 30 min or 37°C for 30 min. A solution about 20 µl dropped on glass object, examined under a fluorescence microscope with 400x magnification, to detect the existence of fluorescence color of greenish yellow from T cell which expressed both CD4 and CD8. If there was fluorescence color of greenish yellow which showed activated immune response, then the counting toward the number of fluorescent T cells would be done.

## Results

**Figure-1:**
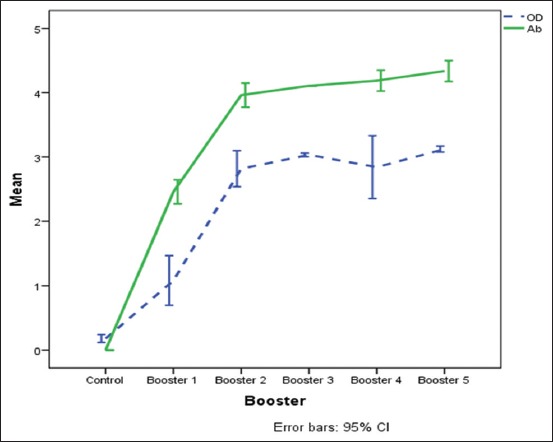
Level of optical density value and antibody titer (immunoglobulin G) of rabbit resulted from immunization with soluble mite protein.

The humoral immune response was shown by OD value enhancement on 3^rd^ booster that reached 16.3 times (mean 3.0424±0.0313) compared to OD control value (mean 0.1838±0.0552). Based on the statistical analysis (multivariate tests), it showed that there was a significant enhancement (p<0.05) for both values from OD value or antibody titer. From the data obtained, the increasing OD value started from day 14 and reached the peak on day 28 (2^nd^ booster), the enhancement stays up to day 42 (3^rd^ booster), and constantly increases up to day 70 or 5^th^ booster (3.1226±0.0430). As well as antibody titer measurement was performed by series of serum dilution then based on cutoff value, it can be known at which dilution antibodies can still form a specific reaction. According to the statistical analysis, it showed that between the control and treatment (booster) group, there is a significant increase (p<0.05) (Figure-1).

**Figure-2:**
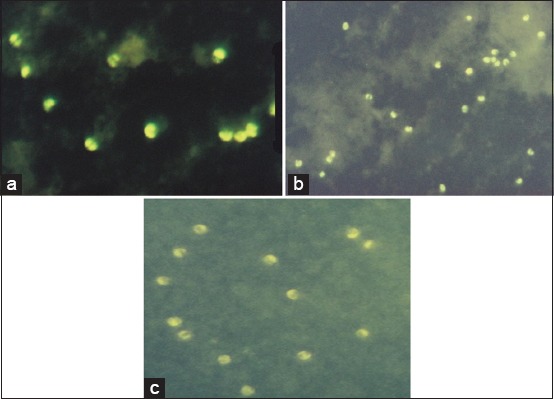
Toll like receptor-9 (a), CD4 (b), CD8 (c) expression in Rabbit T cells, visualized by fluorescein isothiocyanate (400× magnification).

The cellular immune response was shown by the expression of TLR-9, CD4, and CD8 in rabbit T cells which is marked by the yellow-to-green fluorescence color after rabbit immunization up to 5 times booster (Figure-2). In addition, the amount of fluorescence T cells was counted for each 20 µl buffy coat; it was shown the amount of fluorescence in T cells is increased in accordance with the treatment from various boosters (booster one up to booster five).

**Figure-3:**
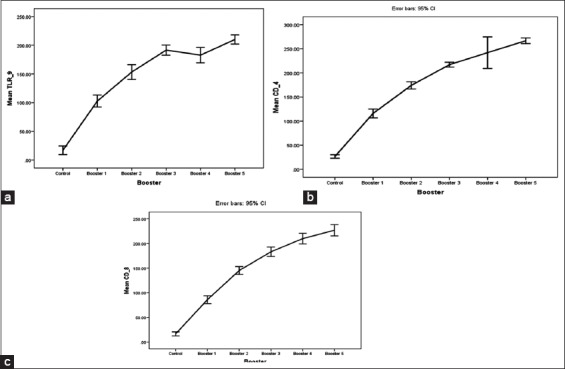
The amount T cells of rabbits which express toll-like receptor9 (a), CD4 (b), CD8) (c), resulted from immunization with soluble mite protein.

The amount of T cells which expresses TLR-9 shows a significant difference (p<0.05) between control and various booster (repetations), but between booster 3 (day 42) and booster 4 (day 56), there is no significant difference by statistic despite the increasing amount of fluorescence T cells, while the amount of T cells which expresses CD4 shows a significant difference (p<0.05) between control and various booster (repetations), as well as the amount of T cells which expresses that CD8 shows a significant difference (p<0.05) between control and various booster as mentioned in Figure-3.

## Discussion

Antibody titer (IgG) enhancement is caused by the memory cells’ activation which happened on the 30^th^ day after initial immunization, and adaptive immune system is like nerve system, capable of memorizing, particularly in experiencing antigen exposure. The immunization result indicated that *S. scabiei* var. *caprae* protein has an ability to induce humoral immune response to produce antibodies (IgG) in immunized rabbits. In accordance with the principles of immunization method, which is increasing the degree of immunity, providing protective immunity by inducing memory response toward specific pathogens with non-virulent or non-toxic antigen [20-23]. In the initial immunization process, there was a recognition by naive T cells and naive B cells; then there was an active phase which was expansive clonal from T cells and differentiated B cells that occurred on day 7 post-immunization [23]. The next process was an antigen elimination or also known as effector phase, this process occurred on day 14, after that apoptosis process occurred and this was followed by the memory cells’ activation in day 30 [20,22]. Adaptive immune system is like the nervous system, capable of memorizing particularly in experiencing antigen exposure; this is the reason why immunity is developed through immunization like the phenomena showed in experimental animal. When the experimental animal immunized by one of antigen, immune response (antibody) or cell-mediated will be appeared after several days, increased rapidly and exponentially (can reach the peak on day 50), then decreased gradually, this is the characteristic of primer immune response [24].

*S. scabiei* var. *caprae* protein has a signal character mediated by TLR9, as well as an immune response induced through CD4 activation related to humoral and CD8 immune responses related to cellular immune responses. This finding is an immune cell communication that plays a role in the dependent pathogenesis of the type of protein expressed in each animal. Based on the exploratory results of the *S. scabiei* var. *caprae* mite protein, it has been demonstrated that the mite contains a protein capable of inducing humoral and cellular immune responses that can be used further research for the development of subunit vaccines for goat scabies.

## Conclusion

Antigenic protein of *S. scabiei* var*. caprae* has the ability to induce humoral and cellular immune response through the enhancement of IgG titer, expression of TLR-9, CD4, and CD8 in rabbits’ T cells. TLR-9 signal is capable not only plays a role in innate immunity but also in adaptive immunity. Antigenic protein of *S. scabiei* var. *caprae* may contain a ligand which acts as a receptor that is involved in the pathogen-associated molecular pattern, and as preliminary research to explore the immunogenic protein which plays a role in the immune system activation for the future studies in vaccine development to overcome scabies on goats.

## Authors’ Contributions

NDRL, WMY, LTS, PH, and DC were carried out the main research works, PH and DC performed the statistical analysis of the main data in the experiments, and all of the authors were approved the final manuscript. All authors read and approved the final manuscript.
